# High Power Ultrasound Treatments of Red Young Wines: Effect on Anthocyanins and Phenolic Stability Indices

**DOI:** 10.3390/foods9101344

**Published:** 2020-09-23

**Authors:** Emilio Celotti, Stefano Stante, Paola Ferraretto, Tomás Román, Giorgio Nicolini, Andrea Natolino

**Affiliations:** 1 Department of Agricultural, Food, Environmental and Animal Sciences, University of Udine, via Sondrio 2/A, 33100 Udine, Italy; emilio.celotti@uniud.it (E.C.); stantestefano@gmail.com (S.S.); patapao2000@yahoo.it (P.F.); 2 Fondazione Edmund Mach—Technology Transfer Center, via Edmund Mach 1, 38050 San Michele all’Adige, Italy; tomas.roman@fmach.it (T.R.); giorgio.nicolini@fmach.it (G.N.)

**Keywords:** red wine, ultrasound irradiation, anthocyanins, phenolic stability, evolution

## Abstract

Polyphenols, especially anthocyanins, play an important role on red wine sensory qualities and their evolution during storage. High Power Ultrasound (HPU) has been recognized as one of the most promising technologies which can be applied in winemaking processes for several purposes, and it is recently officially approved for crushed grapes treatments. The effect of ultrasound amplitude (41 and 81%) and treatment time (1, 3, and 5 min) has been studied on anthocyanins, flavan-3-ols, tannins, polymerized pigments, HCl index, and the color intensity of two finished red young wines. Anthocyanins and phenols compounds were not degraded with an increase in amplitude and sonication time, and the chromatic properties of the selected wines were preserved. Amplitude and ultrasound time were also evaluated considering their effect on evolution of anthocyanin content and phenolic stability indices during the first thirty days of storage. The higher level of amplitude (81%) induced a higher percentage decrease in tannins, 15% and 40% after 15 and 30 days of storage, respectively, compared to untreated wine which did not show a significant change during storage. HPU shows a possible chemical effect on the evolution of some analytical indices during bottling maturation, but their effectiveness could be strictly linked with the initial phenolic profile and ratios between polyphenol classes.

## 1. Introduction

Wine aging is a central process with the aim to achieve stable and high-quality wines. Many changes occur in the composition of the wine during this period, accompanied by the development of color, aroma and flavor.

The evolution of color, especially in red wines, represents a key role during the aging process [1]. Generally, color is thought as a basis for the assessment of quality. Based on this point, color plays an important role in sensory evaluation and consumers’ expectation on foods and drinks, which gives a visual guide to the taste and flavor sensory characteristics, influencing preference, acceptability and final choice [2,3]. To some extent, the wine color can reflect the wine quality, and consumers also tend to prefer wines with deeper color [4]. Color depends on many variables present throughout all the vinification processes, such as the grape varieties, wine-making techniques, and stabilization and aging procedures employed [5].

During storage and bottle aging, the color of red wines turns deeper and more stable, and changes from a bright red to a reddish-brown tonality [6]. The color and its evolution are determined by many variables but is related to the phenolic compounds in the wine, particularly anthocyanins, which undergo to copigmentation reaction with other compounds and progressive formation of new pigments [7,8]. Copigmentation occurs as a result of anthocyanins ability to form associations between themselves or with copigments based on weak interactions, mainly Van der Walls or hydrophobic forces. Different molecules have been proposed to act as copigments, from monomeric anthocyanin themselves to phenolic acids, flavonols, tannins or other compounds [9,10]. The magnitude of copigmentation strongly depends on several factors, such as nature and structure of copigment, pH, transition-metal, ethanol concentration, acetaldehyde, and others [11].

Wine aging is a high-cost and long term process, which is sometimes incompatible with the consumers’ requirements. Several actions should be considered to accelerate the vinification processes and to ensure the quality of the wine at the same time. Different physical technologies, such as ultrasound [12,13], gamma irradiation [14] and high pressure [15,16], have been applied to accelerate the aging process and shorten the production cycle of the wine.

Ultrasound processing is a relatively low-cost, non-hazardous, eco-friendly and non-thermal processing technology, commonly applied in food industries for several purposes [17].

In wine research, this technology has been applied for several purposes in winemaking processes, such as the extraction of phenolic compounds during maceration [18], the extraction of aroma from must and wine [19], replacing the preservatives addition [20], management of wine microbiology [21], and valorization of winery by-products [22,23]. In addition, ultrasound is also regarded as the most promising technique for accelerating the wine aging process [24,25], specifically considering its effectiveness on changing the chromatic characteristics and phenolic properties of red wines [16,26,27,28].

In 2019, the International Organization of Wine (OIV) has officially recognized the effectiveness of ultrasounds through the release of a specific resolution concerning the application of ultrasounds on crushed grapes to promote the release of their compounds, specifically polyphenols [29].

Taking into account of the recent OIV resolution and the potential chemical effect, the aim of this work was to evaluate the effect of ultrasounds on anthocyanins’ stability and their evolution along maturation time. Considering the results reported in a previous work [27], the effect of ultrasound irradiation at different amplitude and sonication time on anthocyanins and analytical indices featuring the color of red wine was considered. In the present study, two red young wines from Valpolicella region were selected due to their low phenolic content, and therefore the maintenance of the initial chromatic characteristics represents a critical aspect of consumers’ acceptance. Moreover, the evolution of the phenolic compounds and phenolic stability indices, during the first thirty days of storage, was monitored to highlight any possible positive effects of ultrasound treatments.

## 2. Material and Methods

### 2.1. Reagents and Solvents

Ethanol, butanol, and methanol were of analytical grade (purity ≥ 99%) and purchased from Sigma-Aldrich Co. (Milan, Italy). The chemicals used, that include hydrochloric acid, formic acid, potassium bisulphite, potassium metabisulphite, 4-(dimethylamino)-cinnamaldehyde, malvidin-3-glucoside and (+)-catechin, were of analytical grade and purchased from Sigma-Aldrich Co. (Milan, Italy).

### 2.2. Wine Samples

Two red wines (A and B) from two different wineries of the Valpolicella region (Italy) were collected and bottled in 100 mL bottles and capped with a crown cap. “Grapes of *Vitis vinifera* var. Corvino and Corvinone were harvested by hand at technical maturity, and were transported immediately at the winery where they were destemmed, crushed and mixed at the same proportions (50:50). An SO_2_ addition at 60 mg/L was made and maceration/fermentation was carried out in stainless steel tanks at 20 °C for 6 days, with a temperature increase until 25 °C. Daily macro oxygenation was carried out injecting oxygen at 10–15 mg/L to promote the color stability. The same selected yeasts were used for both red young wines. At the end of six days of maceration, the wine is racked, the grape marc was soft pressed, and the liquid was added to the previous racked wine. At the end of alcoholic fermentation, malolactic fermentation was carried out spontaneously. The wine was stored and aged in stainless steel tanks at 15 °C and no barrel aging was provided. Sulfites content was maintained at 15 mg/L of free sulfur dioxide.

The chemical characteristics of the selected wines (Table 1) were determined according to the official methods of the International Organization of Vine and Wine [30].

### 2.3. Ultrasound Treatments

All the experiments were carried out in an ultrasonic sonifier (Sonoplus model HD 2200, Bandelin electronic, Berlin, Germany) equipped with a titanium alloy flat tip probe (13 mm diameter) (TT13, Bandelin, Berlin, Germany). Samples were processed in a continuous sonication at a constant frequency of 20 kHz. The energy input was controlled by setting the amplitude of the sonicator probe; the total nominal output was 200 W. The ultrasound probe was submerged to a depth of 20 mm in a 250 mL beaker containing the sample. The beaker was placed into an ice bath to avoid the increase in temperature of up to 35 °C, which was continuously monitored using a temperature controller.

The experimental conditions of ultrasound treatments were set considering previous research works, and they are generally applied on wineries for the treatment of red crushed grapes [27,31]. Samples of wine A (WA) were sonicated for 3 min at two levels of amplitude (41% and 81%) to assess the amplitude effect. Instead, samples of wine B (WB) were sonicated at a fixed amplitude (81%) but at different levels of sonication time (t_US_) (0, 1, 3, and 5 min). The experimental conditions are summarized in Table 2. All the experiments were carried out in triplicate.

### 2.4. Analytical Methods

All analytical parameters were measured at different times (0, 15, and 30 days) after ultrasound (US) treatments to evaluate the sonication effect on wine aging.

#### 2.4.1. Anthocyanins Content

Anthocyanins content was determined as reported by Ribereau Gayon and Stonestreet [32]. One milliliter of sample was mixed with 1 mL of HCl/EtOH solution (0.1% *v*/*v*) and 20 mL of HCl/H_2_O solution (2% *v*/*v*). Then, 2.5 mL of sample mixture was added with 1 mL of deionized H_2_O and other 2.5 mL of sample mixture with 1 mL of potassium bisulphite solution (20% *w*/*v*). After 15 min of reaction, the absorbance of each solution was measured at 520 nm using an UV-Vis spectrophotometer (Shimadzu UV 1650, Milano, Italy), using distilled water as a control. The anthocyanin content, expressed as milligrams of malvidin-3-glucoside equivalent per liter (mg/L), was calculated considering a calibration curve, obtained with different solutions of malvidin-3-glucoside as standard. All the determinations were performed in triplicate.

#### 2.4.2. Color Intensity (C.I.)

Color intensity was determined by spectrophotometer measurements of undiluted samples on a 1 mm optimal path [33]. The optical densities were measured in a UV-Vis spectrophotometer (Shimadzu UV1650, Milano, Italy) at 420, 520 and 620 nm. Color intensity (CI) was calculated as follow:

CI=OD420+OD50+OD620

Results were calculated considering a final optical path of 10 mm. All the measurements were performed in triplicate.

#### 2.4.3. Flavan-3-ols Content

Flavan-3-ols content was determined according to the method proposed by Zironi et al. [34]. The chromogen reagent was prepared with 1 g of 4-(dimethylamino)-cinnamaldehyde (DAC) dissolved into 250 mL of 37% HCl and 750 mL of methanol. Next, 1 mL of diluted sample (1:25 *v*/*v*) was added to 5 mL of DAC solution. Then, absorbance was read at 640 nm in a UV-Vis spectrophotometer (Shimadzu UV 1650, Tokyo, Japan) against a blank prepared by substituting the sample with 1 mL of 10% ethanol solution. A calibration curve was made with several standard solutions of (+)-catechin and measurements were carried out at 640 nm. All analyses were performed in triplicate. Results were expressed as milligrams of (+)-catechin equivalents per liter (mg/L).

#### 2.4.4. Condensed Tannins

Condensed tannins content was determined following the method reported by Bate-Smith [35]. two reaction mixtures were prepared mixing 2 mL of diluted sample and 6 mL of hydrochloric acid-butanol solution. One of reaction mixture was placed in water bath at 100 °C for 20 min. Subsequently, absorbance at 550 nm was measured in a UV-Vis spectrophotometer (Shimadzu UV 1650, Tokyo, Japan). The concentration of condensed tannins was then calculated by the equation:

$$TA={∆}_{Abs}\cdot DF\cdot 0.1736$$

where *TA* is tannins concentration, expressed as grams per liter (g/L), and *DF* is dilution factor. All analyses were performed in triplicate.

#### 2.4.5. Polymerized Pigments Index (PPI)

The Polymerized Pigments Index (PPI) was determined as reported by Glories [36]. PPI represents the contribution to the red color of condensed tannins and polymerized form of anthocyanins insensitive to bleaching by sulfur dioxide. Two reaction solution were prepared as follow: (A) 5 mL of sample was mixed with 45 mL of tartaric buffer (pH 3.2) and 0.2 mL of potassium metabisulfite solution (20% *w*/*v*); (B) 5 mL of sample was mixed with 45 mL of tartaric buffer (pH 3.2) and 0.2 mL of water. After 5 min, the optical density of each solution was read at 420 and 520 nm in a UV-Vis spectrophotometer (Shimadzu UV 1650, Tokyo, Japan), against water as blank. Polymerized Pigments Index was then calculated by the equation:

$$PPI=\;\frac{OD420_{\left(A\right)}+OD520_{\left(A\right)}}{OD420_{\left(B\right)}+OD520_{\left(B\right)}}$$

All analyses were perfomed in triplicate.

#### 2.4.6. HCl Index

HCl index was measured following the method described by Glories [36]. The method is based on the instability of procyanidins in concentrated HCl medium, and their speed of precipitation depends on the degree of polymerization. Sample solutions were prepared by mixing 10 mL of sample, 15 mL of 12 N HCl and 5 mL of water. The sample solution was diluted 25 times its original volume, and the optical density (d_0_) at 280 nm was measured immediately in a UV-Vis spectrophotometer (Shimadzu UV1650, Tokyo, Japan). The same measurement of optical density at 280 nm (d_1_) was performed after waiting 24 h and centrifuging the mixture at 3000× *g* × 10 min. HCl index is given by the equation:

$$I\left(HCl\right)=\;\frac{\left(d_{0}-d_{1}\right)}{d_{0}}\times 100$$

All the determinations were carried out in triplicate.

#### 2.4.7. Analysis of Anthocyanins by High Performance Liquid Chromatography (HPLC)

HPLC analyses were performed on a LC-2010 AHT liquid chromatographic system (Shimadzu, Kyoto, Japan), equipped with an integrated autosamplet and UV-Vis detector. Compounds were separated on a 5 μm packed, 150 × 4.6 mm Zorbax Eclipse Plus C18 column (Agilent Technologies, Santa Clara, CA, USA) thermostated at 25 °C. The elution was performed in a gradient mode with a flow rate of 1.2 mL/min. The mobile phase was composed of 9% (*v*/*v*) formic acid in Milli Q grade water (solvent A) and 9% (*v*/*v*) formic acid in HPLC grade methanol (Solvent B). The gradient was set as follows: solvent B was held at 10% for the first 3 min, increased to 50% in the following 15 min and held at the 50% for additional 2 min. Solvent B was then decreased in 1 min to the initial conditions (10%) and equilibrated at 10% for 2 min. The injection volume was 20 μL. Before injection, all samples were filtered on 0.20 μm nylon membranes (Albet-Hahnemühle, Barcelona, Spain). Detection was performed at 525 nm. Peak identification was based on the order of elution reported in literature [37].

Results are expressed as relative percentage, which was calculated on the peak area of an anthocyanin species to the total peak area of all the anthocyanins in the wine sample. Each result presents the mean and the standard deviation for a minimum of three analyses.

### 2.5. Statistical Analysis

All experiments were performed in triplicate, and all results were expressed as mean ± standard deviation. Minitab 17 software (Minitab Inc., State College, PA, USA) was used for statistical analysis by one-way analysis of variance (ANOVA, with Tukey’s HSD multiple comparison) with the level of significance set up at *p* ≤ 0.05.

## 3. Results and Discussion

### 3.1. Effect of Ultrasound Amplitude

The experimental values of anthocyanins, flavan-3-ols, tannins content, polymerized pigments index, HCl index and color intensity of untreated (WA) and sonicated wine at different amplitudes (WA-41 and WA-81) are shown in Table 3.

It can be observed that no significant differences can be highlighted between untreated and sonicated samples for anthocyanins, tannins, P.P.I., HCl index, and color intensity before bottling maturation (t_stor._ = 0). The increase in ultrasound amplitude from 41% to 81% induced a significant decrease in favan-3-ols content, from 182.66 ± 11.96 to 158.03 ± 6.00 mg/L, which could undergo to chemical degradation promoted by ultrasounds as reported by Zhu et al. [38].

Despite flavan-3-ols’ decrease, the sonication conditions adopted in the present work permits to preserve the anthocyanin content and chromatic properties, at t_stor._ = 0. As reported by Lukic et al. [13], the application of ultrasound should ensure the preservation of sensory properties of wines, including color characteristics and stability. Ultrasonic waves could lead to changes in phenolic composition due to cavitation phenomenon, which can induce the formation of free radical species and trigger oxidation reactions in wine. High levels of amplitude generates high intense acoustic cavitation, which can induces degradation of phenol compounds [39]. In view of this, it is extremely important to select suitable ultrasound amplitudes to preserve polyphenols content and chromatic properties, mostly for red wines of Valpolicella region which are characterized by low anthocyanin contents and color intensities.

Following the ultrasound treatments, samples were bottled and their chromatic properties and phenolic compounds were monitored after 15 and 30 days of storage (Table 3). Common trends of the analytical indices during wine storage can be observed for all treatments: a decrease in anthocyanin and tannins content, and an increase in flavan-3-ols, polymerized pigments and HCl index.

During wine aging, several copigmentation and stabilization reactions can occur between anthocyanin and tannins, resulting in their progressive disappearance and the formation of new pigments with a higher degree of polymerization [7,40]. The decrease in tannins may also be related to the increase in flavan-3-ols content, as tannins can undergo a depolymerization phenomenon, resulting in an increase in monomeric units such as catechins [41]. This could be favored by ultrasound waves, which could have promoted the depolymerization and recombination reactions of phenolic compounds [28] after an initial lowering of the flavan-3-ols in treated wine samples.

Concerning color intensity (C.I.), no changes were observed during storage for untreated samples at t_stor._ = 0 (2.63 ± 0.11), after 15 days (2.83 ± 0.11) and 30 days (2.70 ± 0.76) of storage. The same happened with treated samples, for which storage did not significantly affect the color intensity, with final values similar to untreated wines.

Despite the common trends observed for untreated and sonicated samples during storage, it is interesting to evaluate the possible effects on the variation rate at which analytical indexes change during 15 and 30 days of storage. The relative percentage variation of anthocyanins, flavan-3-ols, tannins, PPI and HCl index for untreated (WA) and sonicated samples (WA-41, WA-81) after 15 and 30 days of storage, compared with the beginning of storage time (t_stor._ = 0), can be considered. It is notable that the application of ultrasound treatment showed a significant increase in variation rate only for tannins, proved by the higher absolute percentage variation when 81% of amplitude was applied—15% and 40% at t_stor._ = 15 and 30 days, respectively. This may be indicative of a positive chemical and amplitude enhancing effect promoted by ultrasound. PPI evolution is favored by sonication only after the first 15 days of storage and no strong differences can be highlighted at t_stor._ = 30 days between untreated and sonicated sample at different amplitudes. Sonication at 41% and 81% of amplitude induces slight higher percentage variations of PPI—7% and 5%, respectively, at t_stor_. = 15 days. As reported by Garcia-Martin et al. [25], acoustic cavitation creates high localized temperatures and pressure, generates certain chemical reactions and accelerates reaction rates. The application of ultrasounds to wine could promote wine compounds interaction and render chemical and structural changes in wine that resemble those occurring after long periods of natural ageing. Sonochemical treatments induce the formation of reactive radical species, mainly ▪OH from the scission of water when it acts as a solvent, which enhances the reaction rates of existing processes or starting new reaction mechanisms [42,43,44,45]. It has been also demonstrated that the sonication of wine and model systems can induce formation of specific free radical species, such as 1-hydroxyethyl free radical [46]. Ultrasound treatments did not significantly affect the percentage variation of anthocyanins and color intensity. Considering the official approval of ultrasound by OIV, the results obtained could indicate that sonication treatments until 81% amplitude could preserve the anthocyanins and, generally, the phenolic compounds, during the treatment of crushed grapes.

An HPLC analysis to measure the main anthocyanins in wine was carried out on untreated and sonicated samples, before bottling maturation (t_stor._ = 0), to highlight any possible effect of ultrasound at different amplitudes on anthocyanin profiles (Table 4). The anthocyanin profile was not affected by sonication treatment and amplitude increase, and no significant differences were observed between samples except for delphinidin-3-monoglucoside, which showed a significant increase from 1.60 ± 0.16% of untreated wine to 3.15 ± 0.26 and 3.21 ± 0.19 of sonicated samples, respectively, at 41% (WA-41) and 81% (WA-81) of amplitude. It is notable that this compound represents only 1–3% of anthocyanin profile, and the main compounds are not affected by ultrasound treatments.

**Figure 1:**
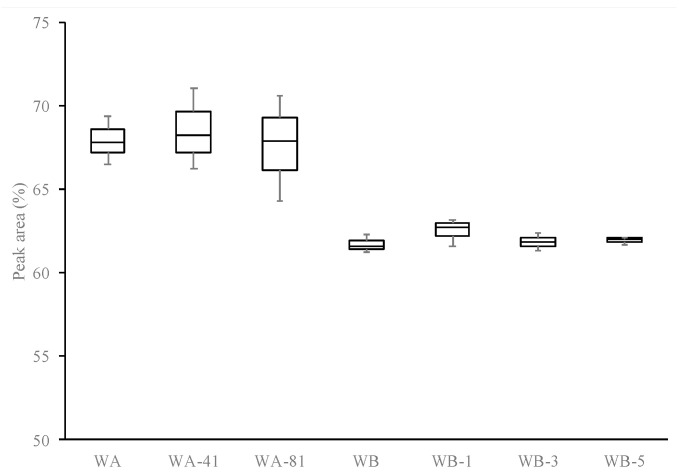
Box-plot of Malvidin-3-monoglucoside percentage on untreated (WA, WB) and sonicated (WA-41, WA-81, WB-1, WB-3, and WB-5) samples.

Indeed, as reported in Figure 1, malvidin-3-glucoside, the main anthocyanin of red wines, is not affected by sonication treatments at 41 (68.51 ± 2.43) and 81% (67.65 ± 3.17) of amplitude, compared to the untreated sample (67.95 ± 1.45).

The same results are reported by Zhang et al. [28], who highlighted no changes on malvidin-3-glucoside content in wine samples after 14 and 28 min of sonication.

### 3.2. Effect of Ultrasound Time (t_US_)

Sonication time (t_US_) represents one of the main parameters to be considered and optimized to obtain the desired targets and, at the same time, to avoid undesirable effects [47]. Anthocyanins, flavan-3-ols, tannins content, polymerized pigments index, HCl index, and color intensity for untreated (WB) and sonicated wine at different irradiation times (WB-1, WB-3 and WB-5) are shown in Table 5. One-way analysis of variance (*p* < 0.05) of free anthocyanins did not show significant differences between the untreated and sonicated samples at the beginning of storage time (t_stor._ = 0). Moreover, the increase in sonication time (t_US_) from 1 (293.27 ± 12.56 mg/L) to 5 min (273.44 ± 1.15 mg/L) did not highlight any significant effect of ultrasound on anthocyanin content.

This is confirmed also by HPLC analysis, the results of which are shown in Table 4 and Figure 1. Qualitative profiles of glucosidic and acetylated anthocyanins did not change, and no significant differences can be highlighted between untreated and sonicated samples, as reported by Cao et al. [48]. Sonication treatments at 81% of amplitude and short times may preserve and not affects the anthocyanin profile of selected red wines. Contrarily, other authors [49] reported that sonication treatments at high amplitude and longer time can promote the degradation of anthocyanins of red grape juice, and their degree of degradation was specific to each individual anthocyanin. Higher sonication times up to 20 min have been tested on commercial red wines and unacceptable limits of chromatic and sensory properties were achieved. As reported by Ferraretto et al. [27], sonication treatment should be accurately modulated to control and manage the quality and stability of wines.

Evaluating flavan-3-ols and tannins contents, color intensity, PPI and HCL index, there are no significant differences between untreated and sonicated samples at the beginning of pre-bottling storage (t_stor_. = 0).

As reported in Table 5, untreated and sonicated samples of wine B show the same evolution of all analytical parameters during storage time. Specifically, anthocyanins and flavan-3-ols content reports a general decrease during 30 days of storage but it is possible to observe a slight increase between 15 and 30 days of bottling. Tannins content decreases during the first 15 days of storage and subsequently increases with no significant differences after 30 days of storage, compared with the beginning (t_stor._ = 0). HCl index shows a significant increase only during the first 15 days of storage, indicating an increase in the polymerization degree of tannins and a potential decrease in wine astringency. Color intensity also increase in the first 15 days, and no variations can be observed at t_stor._ = 30 days. PPI index achieve a maximum content at t_stor._ = 15 days, when a minimum values of anthocyanins and tannins were revealed. These effects may be may be due to correlation between the anthocyanin and tannins, and their involvement in copigmentation reactions that occur with a formation of new pigments and an increase in color intensity. In view of these results, sonication treatments could preserve not only the initial phenolic and chromatic characteristics of the wine but also their evolution during storage.

As reported above, the effect of ultrasound time on polyphenols and stability indices evolution during bottling storage can be preliminarily evaluated considering the absolute percentage variation of analytical parameters. The percentage variation of anthocyanins, flavan-3-ols, tannins, color intensity and HCl index for untreated (WB) and sonicated samples (WB-41, WB-81) after 15 and 30 days of storage, compared with the beginning of storage time (t_stor_. = 0 have not highlighted significant changes at different ultrasound times. Nevertheless, anthocyanins, tannins, PPI index and color intensity show a decrease trend of absolute percentage variation with an increase in sonication time. This behavior may indicate that long sonication treatments negatively affect the wine evolution processes. Ultrasound irradiation can promote the degradation of chemical compounds, but, moreover, can induce radical species formation which may give rise to other chemical reactions [48]. Instead, no possible effect can be highlighted for the HCl index and tannins content.

Comparing the evolution of untreated wine A and wine B, obtained at the same sonication treatments, at 81% of amplitude and 3 min of sonication time (sample WA-81 and WB-3), different trends of analytical parameters can be seen. Differences in the evolution processes can be attributed to the initial chemical and physical properties of the wine. Aging process are affected by several factors, such as: pH, ethanol concentration, grape variety, winemaking process, anthocyanins and flavanols concentration, etc. [49,50]. Specifically, an important role is played by the ratios between different classes of phenolic compounds, such as tannins and anthocyanins (T/A ratio), which can affect the color and phenolic development of red wines [51]. As can be noted, wine A shows an higher T/A ratio than wine B—8.17 and 4.58, respectively, indicating a higher ability of wine A to achieve better evolution and stabilization reactions of phenolics and chromatic properties.

It is of critical importance to apply the ultrasound treatments in relation to the initial phenolic profile, in order to avoid any undesirable effects.

## 4. Conclusions

Taking into account the potential of ultrasound technology in winemaking processes, the effect of amplitude and sonication time on anthocyanins and phenolic stability indices were analyzed on two different red young wines. Results showed that sonication treatments can preserve polyphenols compounds and not induce degradation reactions, a fundamental aspect when the initial phenolic profile is low and the preservation of chromatic and sensory properties becomes of paramount importance.

Ultrasound treatments should be accurately modulated depending on the physical and chemical properties of the wine, particularly the ratio of different classes of phenolic compounds. A potential positive chemical effect of ultrasound on evolution of some analytical indices, related to polyphenols stability and their evolution, has been highlighted, but it requires more detailed investigations, especially on the polymerization and co-pigmentation reactions of anthocyanin and tannins.

Moreover, in view of the official approval by OIV on the application of ultrasound technology on crushed grapes, further research is needed to deeper investigate the effect of ultrasound treatments on anthocyanins and other phenolic compounds, with the aim to enhance the extraction efficiency from grapes but otherwise to preserve their chemical structures.

## Figures and Tables

**Table 1:** Chemical properties of selected red wines.

Wine	Alcohol (%)	pH	Total Acidity (g L^−1^)	Free SO_2_ (mg L^−1^)	Total SO_2_ (mg L^−1^)
WA	11.5	3.72	5.20	20	60
WB	11.5	3.23	8.35	29	55

**Table 2:** Experimental ultrasound conditions.

Wine	Sample Code	Amp (%)	t_US_ (min)
A	WA *	0	3
	WA-41	41	3
	WA-81	81	3
B	WB ^#^	81	0
	WB-1′	81	1
	WB-3′	81	3
	WB-5′	81	5

* WA (Wine A untreated), WA-41 WA-81 (Wine A sonicated at 41% and 81% of amplitude). ^#^ WB (Wine B untreated); WB-1′, WB-3′ and WB-5′ (Wine B sonicated for 1, 3 and 5 min).

**Table 3:** Effects of ultrasound amplitude and storage time on anthocyanins, flavan-3-ols, tannins content, polymerized pigments index (P.P.I.), HCl index and color intensity (C.I.) of red wine A.

Sample	Amplitude	t_stor._	Anthocyanins	Flavan-3-ols	Tannins	P.P.I.	HCl Index	C.I.
	(%)	(day)	(mg/L)	(mg/L)	(g/L)	(-)	(-)	(-)
WA	0	0	185.99 ± 1.58 a *	201.07 ± 7.13 c	1.52 ± 0.16 ab	45.61 ± 1.09 c	8.82 ± 0.73 c	2.63 ± 0.11 a
	0	15	169.27 ± 3.43 b	227.94 ± 3.92 b	1.55 ± 0.09 ab	45.65 ± 0.60 c	18.22 ± 1.01 b	2.83 ± 0.11 a
	0	30	143.86 ± 3.32 c	283.02 ± 3.22 a	1.42 ± 0.04 b	53.75 ± 0.55 ab	33.73 ± 0.90 a	2.70 ± 0.76 a
WA-41	41	0	188.88 ± 3.52 a	182.66 ± 11.96 c	1.54 ± 0.52 ab	45.31 ± 0.61 c	8.62 ± 1.28 c	3.07 ± 0.48 a
	41	15	168.23 ± 3.76 b	224.78 ± 7.22 b	1.47 ± 0.43 ab	48.63 ± 2.96 bc	18.32 ± 6.47 b	3.10 ± 0.24 a
	41	30	144.70 ± 4.72 c	279.08 ± 8.99 a	1.23 ± 0.40 ab	54.52 ± 0.87 a	36.30 ± 1.98 a	2.94 ± 0.07 a
WA-81	81	0	194.10 ± 2.50 a	158.03 ± 6.00 d	1.67 ± 0.08 a	46.46 ± 3.87 c	7.81 ± 2.48 c	2.89 ± 0.18 a
	81	15	166.54 ± 5.80 b	234.48 ± 9.30 b	1.17 ± 0.14 b	48.69 ± 2.90 bc	20.89 ± 2.94 b	3.14 ± 0.05 a
	81	30	137.11 ± 9.78 c	285.40 ± 5.38 a	1.01 ± 0.06 c	53.05 ± 1.28 ab	33.38 ± 2.53 a	2.97 ± 0.04 a

* Each datum represents the mean of three replicates ± standard deviation. Values with different letter within column indicate significative differences (*p* < 0.05).

**Table 4:** Minor anthocyanins’ composition (%) of wine A and B, respectively, at different amplitudes (WA, WA-41, WA-81) and sonication times (WB, WB-1, WB-3 and WB-5), at t_stor._ = 0.

Compound	Wine A			Wine B			
	WA	WA-41	WA-81	WB	WB-1	WB-3	WB-5
Delphinidin-3-monoglucoside	1.60 ± 0.16 c *	3.15 ± 0.26 b	3.21 ± 0.19 b	3.97 ± 0.36 a *	3.94 ± 0.30 a	3.88 ± 0.24 a	4.15 ± 0.15 a
Cyanidin-3-monoglucoside	0.46 ± 0.08 b	0.68 ± 0.15 b	0.69 ± 0.11 b	0.98 ± 0.20 a	0.93 ± 0.18 a	0.95 ± 0.20 a	0.99 ± 0.02 a
Petunidin-3-monoglucoside	5.03 ± 0.23 a	5.00 ± 0.21 a	4.96 ± 0.24 a	5.51 ± 0.29 a	5.10 ± 0.78 a	5.52 ± 0.14 a	5.30 ± 0.33 a
Peonidin-3-monoglucoside	7.65 ± 0.39 b	9.88 ± 1.25 a	9.55 ± 0.55 a	10.39 ± 0.02 a	10.75 ± 0.12 a	10.30 ± 0.65 a	10.94 ± 0.28 a
Vitisin A	1.14 ± 0.10 b	1.32 ± 0.11 b	1.33 ± 0.11 b	2.17 ± 0.07 a	2.13 ± 0.16 a	2.27 ± 0.10 a	2.19 ± 0.08 a
Petunidin-3-monoglucoside acetylated	0.55 ± 0.09 b	0.66 ± 0.11 b	0.55 ± 0.06 b	0.73 ± 0.03 a	0.71 ± 0.05 a	0.74 ± 0.07 a	0.69 ± 0.02 a
Peonidin-3-monoglucoside acetylated	1.35 ± 0.17 a	1.34 ± 0.38 a	1.91 ± 0.44 a	2.12 ± 0.36 a	1.64 ± 0.68 a	2.00 ± 0.16 a	1.41 ± 0.59 a
Malvidin-3-monoglucoside acetylated	7.37 ± 0.33 a	7.99 ± 1.40 a	7.01 ± 0.37 a	6.39 ± 0.59 ab	6.15 ± 0.58 ab	6.41 ± 0.20 b	6.12 ± 0.12 b
Delphinidin-3-monoglucoside p-coumarylated	2.82 ± 0.16 a	2.48 ± 0.20 a	2.57 ± 0.40 a	2.21 ± 0.10 a	2.25 ± 0.07 a	2.14 ± 0.09 a	2.23 ± 0.05 a
Malvidin-3-monoglucoside p-coumarylated	4.13 ± 0.26 a	4.26 ± 0.26 a	4.39 ± 0.51 a	3.24 ± 0.12 a	3.32 ± 0.05 a	3.37 ± 0.03 a	3.43 ± 0.13 a
Malvidin-3-monoglucoside vinylphenol	n.d. #	n.d.	n.d.	0.15 ± 0.02 a	0.16 ± 0.03 a	0.19 ± 0.01 a	0.18 ± 0.01 a
Malvidin-3-monoglucoside vinylphenol acetylated	n.d.	n.d.	n.d.	0.41 ± 0.01 a	0.37 ± 0.04 a	0.39 ± 0.02 a	0.39 ± 0.03 a

* Each datum represents the mean of three replicates ± standard deviation. Values with different letters within line indicate significative differences (*p* < 0.05). # n.d.= not detected.

**Table 5:** Effect of ultrasound time and storage time on anthocyanins, flavan-3-ols, tannins content, polymerized pigments index (P.P.I.), HCl index, and color intensity (C.I.) of red wine B.

Sample	Time US	t_stor._	Anthocyanins	Flavan-3-ols	Tannins	P.P.I.	HCl Index	C.I.
(min)	(day)	(mg/L)	(mg/L)	(g/L)	(-)	(-)	(-)
WB	0	0	280.99 ± 9.71 a *	298.50 ± 0.72 bc	1.26 ± 0.11 ab	57.87 ± 4.61 b	14.22 ± 0.54 b	4.52 ± 0.06 c
		15	132.18 ± 11.87 c	246.10 ± 0.86 e	1.09 ± 0.07 b	68.95 ± 3.72 ab	28.51 ± 2.40 a	5.55 ± 0.04 a
		30	182.12 ± 3.52 b	262.23 ± 7.43 e	1.08 ± 0.25 ab	60.57 ± 2.96 ab	30.98 ± 1.45 a	5.38 ± 0.17 ab
WB-1	1	0	293.27 ± 12.56 a	300.70 ± 6.22 b	1.34 ± 0.04 a	61.37 ± 1.04 ab	13.13 ± 2.48 b	4.54 ± 0.01 c
		15	133.67 ± 15.74 c	251.03 ± 10.38 e	1.09 ± 0.12 b	71.89 ± 1.97 a	27.66 ± 1.39 a	5.42 ± 0.14 ab
		30	183.02 ± 4.70 b	268.38 ± 2.78 de	1.10 ± 0.24 ab	60.45 ± 2.49 ab	24.27 ± 2.39 a	5.50 ± 0.06 ab
WB-3	3	0	283.68 ± 5.14 a	299.18 ± 9.85 bc	1.26 ± 0.14 ab	67.55 ± 12.73 ab	13.68 ± 2.43 b	4.65 ± 0.01 c
		15	150.85 ± 10.23 c	312.95 ± 26.55 ab	1.05 ± 0.23 b	68.11 ± 0.75 ab	24.74 ± 3.45 a	5.47 ± 0.03 ab
		30	187.25 ± 4.61 b	271.50 ± 3.64 cde	1.51 ± 0.18 a	59.12 ± 0.97 b	25.16 ± 2.44 a	5.33 ± 0.25 ab
WB-5	5	0	273.44 ± 1.15 a	292.75 ± 0.70 bcd	1.19 ± 0.17 ab	64.53 ± 1.87 ab	12.59 ± 1.08 b	4.68 ± 0.11 c
		15	150.33 ± 8.22 c	331.35 ± 4.70 a	0.98 ± 0.17 b	63.14 ± 0.82 ab	26.20 ± 3.38 a	5.32 ± 0.12 ab
		30	190.28 ± 4.11 b	269.73 ± 7.46 de	1.32 ± 0.11 ab	56.87 ± 1.73 b	29.84 ± 4.38 a	5.18 ± 0.11 b

* Each datum represents the mean of three replicates ± standard deviation. Values with different letter within column indicate significative differences (*p* < 0.05).
